# The Prevalence and Risk Factors for Pneumococcal Colonization of the Nasopharynx among Children in Kilifi District, Kenya

**DOI:** 10.1371/journal.pone.0030787

**Published:** 2012-02-20

**Authors:** Osman Abdullahi, Angela Karani, Caroline C. Tigoi, Daisy Mugo, Stella Kungu, Eva Wanjiru, Jane Jomo, Robert Musyimi, Marc Lipsitch, J. Anthony G. Scott

**Affiliations:** 1 Wellcome Trust Research Program, Kenya Medical Research Institute, Centre for Geographic Medicine Research - Coast, Kilifi, Kenya; 2 Department of Immunology and Infectious Diseases, Harvard School of Public Health, Boston, Massachusetts, United States of America; 3 Department of Epidemiology, Harvard School of Public Health, Boston, Massachusetts, United States of America; 4 Nuffield Department of Clinical Medicine, Oxford University, John Radcliffe Hospital, Oxford, United Kingdom; Columbia University, United States of America

## Abstract

**Background:**

Pneumococcal conjugate vaccines (PCV) reduce nasopharyngeal carriage of vaccine-serotype pneumococci but increase in the carriage of non-vaccine serotypes. We studied the epidemiology of carriage among children 3–59 months old before vaccine introduction in Kilifi, Kenya.

**Methods:**

In a rolling cross-sectional study from October 2006 to December 2008 we approached 3570 healthy children selected at random from the population register of the Kilifi Health and Demographic Surveillance System and 134 HIV-infected children registered at a specialist clinic. A single nasopharyngeal swab was transported in STGG and cultured on gentamicin blood agar. A single colony of pneumococcus was serotyped by Quellung reaction.

**Results:**

Families of 2840 children in the population-based sample and 99 in the HIV-infected sample consented to participate; carriage prevalence was 65.8% (95% CI, 64.0–67.5%) and 76% (95% CI, 66–84%) in the two samples, respectively. Carriage prevalence declined progressively with age from 79% at 6–11 months to 51% at 54–59 months (p<0.0005). Carriage was positively associated with coryza (Odds ratio 2.63, 95%CI 2.12–3.25) and cough (1.55, 95%CI 1.26–1.91) and negatively associated with recent antibiotic use (0.53 95%CI 0.34–0.81). 53 different serotypes were identified and 42% of isolates were of serotypes contained in the 10-valent PCV. Common serotypes declined in prevalence with age while less common serotypes did not.

**Conclusion:**

Carriage prevalence in children was high, serotypes were diverse, and the majority of strains were of serotypes not represented in the 10-valent PCV. Vaccine introduction in Kenya will provide a natural test of virulence for the many circulating non-vaccine serotypes.

## Introduction

Pneumococcal disease is estimated to cause 826,000 deaths among young children in developing countries and the majority of these deaths occur in sub-Saharan Africa [1]. By comparison with American children, African children have a higher incidence of invasive pneumococcal disease (IPD) [2], experience disease at an earlier age [3] and are affected by different serotypes, particularly types 1 and 5 [4], [5]. Nasopharyngeal carriage prevalence in unvaccinated children is also higher in African ranging from 48%–90% [6], [7], [8] compared to 35–52% in the UK/USA [9], [10].

Pneumococcal conjugate vaccines (PCV) have substantially reduced the incidence of IPD in children in the USA [11] and have been shown in clinical trials to be effective in African children, including those infected with HIV [12], [13]. In operational use the effectiveness of these vaccines has been offset by an increase in the rate of IPD caused by serotypes not included in the vaccine [14]. In most settings this serotype replacement disease has been small by comparison with the reduction in disease caused by vaccine serotypes. However, in some populations, such as Alaska Native children [15], it has been more substantial.

In 2011, Kenya introduced a 10-valent pneumococcal conjugate vaccine (PCV10) into the national childhood immunization schedule. In Kilifi, prior to vaccine introduction, we established a system of surveillance for childhood IPD to monitor the impact of vaccine introduction on disease. To further understand the effect of vaccine on carriage and transmission we also set up studies to measure the prevalence, rate of acquisition and the rate of clearance from the nasopharynx. Here we describe the serotype-specific prevalence of carriage and the epidemiological risk factors for prevalent carriage in the pre-vaccine era.

## Methods

### Study population

The study was conducted among children aged 3–59 months who were registered residents of the Kilifi Health and Demographic Surveillance System (KHDSS) [16]. This is a longitudinal surveillance of the population living in a well-defined geographic area around Kilifi District Hospital (KDH), which is updated through household visits, monitoring vital events and migration, every four months. At the planning of the present study (1^st^ July 2006) the KHDSS area had a population of 41,651 aged 3–59 months.

### Study design

The study was a rolling cross sectional survey conducted over a period of 26 months and was linked to a study of carriage clearance [17]. As the objective was to define the characteristics of carriage at serotype-specific level, we aimed to recruit sufficient children to observe at least 30 carriers of each serotype that was responsible for colonizing at least 1% of carriers. Estimating pneumococcal carriage prevalence in this age group at 75%, the sample size was 4000 children. Each week we selected a small area within the KHDSS and applied a random number to each child in the population register for that area. We then selected the 10% of children with the highest ranked numbers to study as a population-based sample. The study moved across contiguous areas to minimize travel for follow-up swabs [17].

HIV infection is a strong risk factor for IPD and is known to modulate the epidemiology of carriage. As HIV prevalence among children in Kilifi is low (∼1%), and HIV testing has not been universally applied across the KHDSS, we selected a group of HIV-infected children directly from the register of the HIV clinic at Kilifi District Hospital. At the clinic HIV-infection status was defined by two rapid tests for HIV antibodies, except for children <18 months of age, where it was defined by PCR. We approached families of consecutive children registered at the clinic and resident in the KHDSS either through home visits or at clinic visits, aiming to recruit 100 children.

Children were excluded from either sample if they were no longer resident in the KHDSS area, if their parent/guardian declined consent, or if the child had an illness that prevented us from taking a nasopharyngeal swab.

### Laboratory methods

The study followed the guidelines of the WHO working group on nasopharyngeal studies of *Streptococcus pneumoniae* [18]. Nasopharyngeal specimens were sampled using Dacron-tipped flexible wire swabs passed via the anterior nares to the posterior nasopharynx. The swab tips were immersed in skim-milk tryptone glucose glycerol (STGG) transport medium, separated from its handle with wire cutters and transported at ambient temperature to the laboratory. Internal quality control of STGG was conducted to assure sterility and the ability of the medium to support pneumococcal growth. Swabs were cultured directly on arrival in the laboratory. STGG samples were vortexed vigorously for 20 seconds, 10 µl was inoculated onto a blood agar plate containing 2.5 mcg/ml gentamicin and incubated overnight at 37°C in 5% CO_2_. Pneumococci were identified by α-hemolysis, optochin sensitivity and presence of capsule. We serotyped one colony per plate using Quellung reaction and polyclonal rabbit antisera (Statens Seruminstitut, Copenhagen, Denmark). Antisera to differentiate 6C from 6A were not available at the time of this study.

### Analysis

The analysis was undertaken in STATA v11.2 (StataCorp, College Station, TX). To detect bias in the execution of the sampling methods, we compared the demographic characteristics of the consenting study participants with the randomly selected sample of KHDSS using χ^2^. Pneumococcal carriage prevalence was estimated from the cross-sectional survey and tested against age and month of study by χ^2^ test for trend. For each population sample a separate multivariable logistic regression model was developed using backward stepwise regression, sequentially excluding variables with p>0.05 on likelihood ratio test.

The Kenya Medical Research Institute/National Ethical Review Committee and The Oxford Tropical Research Ethics Committee approved the study. On behalf of minors/children participants involved in this study, written informed consent was obtained from their next of kin, carers or guardians.

## Results

The cross-sectional survey of the population-based sample began October 23, 2006 and ended December 2, 2008. We selected 4,294 children at random from the KHDSS population register and were able to find 3570 (83%) at home. The main reasons for failing to identify children were the high out-migration rate in the KHDSS [16], seasonal migration for farming, and the inability of the study to sample children out of hours. Of the 3570 families located and invited to participate, 2840 (80%) consented to the enrolment of their child into the study. Children who were recruited to the study differed from those who were originally selected on the month of sampling and location of residence but not on age or sex (Table 1). The only prior use of pneumococcal conjugate vaccine (7-valent PCV) in the KHDSS area was a study of 300 children vaccinated as infants [19]; 14 of these were recruited to the present study at ages varying between 4–34 months.

**Table 1:** Demographic distribution of the target population and the study sample.

		Number selected	Included in the study	% Included in the study	χ^2^ p-value
Sex					
	Male	2143	1435	67.0	
	Female	2151	1405	65.3	0.255
Age (months)					
	3–23	1588	1023	64.4	
	24–41	1324	886	66.9	
	42–59	1382	931	67.4	0.184
Location of residence					
	Banda ra salama	145	99	68.3	
	Chasimba	275	208	75.6	
	Gede	168	138	82.1	
	Jaribuni	71	45	63.4	
	Junju	470	280	59.6	
	Kauma	128	102	79.7	
	Kilifi Township	580	293	50.5	
	Matsangoni	341	240	70.4	
	Mtwapa	168	132	78.6	
	Ngerenya	329	249	75.7	
	Roka	381	253	66.4	
	Sokoke	208	136	65.4	
	Takaungu-Mavueni	380	244	64.2	
	Tezo	399	245	61.4	
	Ziani	251	176	70.1	<0.0005
Month of sampling					
	Jan	308	216	70.1	
	Feb	409	278	68.0	
	Mar	392	225	57.4	
	Apr	347	220	63.4	
	May	529	278	52.6	
	Jun	458	283	61.8	
	Jul	353	271	76.8	
	Aug	155	118	76.1	
	Sep	300	178	59.3	
	Oct	435	326	74.9	
	Nov	494	337	68.2	
	Dec	114	110	96.5	<0.0005
Total		4294	2840	66.1	

Children were recruited to the HIV-infected sample between January 14, 2008 and July 21, 2008. Of 134 parents/guardians approached, 99 (74%) consented for their children to participate. There were no differences between those who did and did not consent with respect to age, sex, location or month of sampling (all p>0.05). The HIV-infected sample did not differ from the population-based sample on age in years (p = 0.58) but did differ on sex; the proportion of males was 50.5% (1435/2840) in the population-based sample and 69% (69/99) in the HIV-infected sample (p<0.0005). None of the HIV-infected children had previously received PCV.

**Figure 1:**
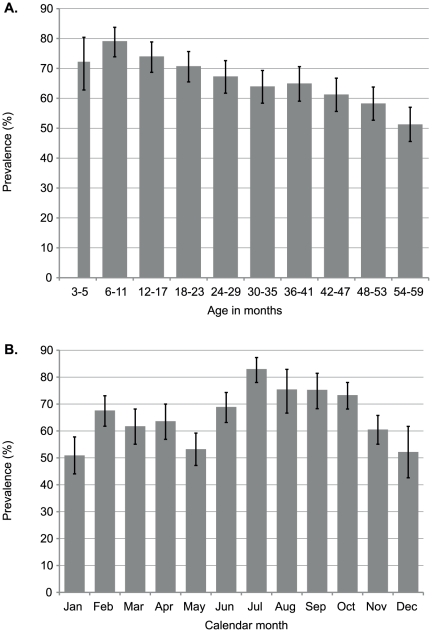
Prevalence (and 95% CI) of nasopharyngeal carriage in children in Kilifi by age and by calendar month within the survey.

Overall, 1868 (65.8%, 95%CI 64.0–67.5%) of the 2840 nasopharyngeal swab cultures from the population-based sample were positive for *Streptococcus pneumoniae*. Carriage prevalence declined markedly with age (χ^2^ test for trend 70.9, p<0.0005) although it was lower in children aged 3–5 months than those aged 6–11 months (Figure 1, Table S2). Carriage prevalence did not vary by sex (p = 0.44, Table S2) but did vary by administrative location across the KHDSS, from 49% to 79% (χ^2^ (14) 84.0, p<0.0005, Table S2), and by month of sampling, from 51% in January to 83% in July (χ^2^ (11) 113.7, p<0.005, Figure 1). The monthly prevalence of carriage was not associated with total monthly rainfall, nor with monthly medians of daily measurements of relative humidity, minimum and maximum daily temperature taken in Kilifi across the 26 months of the study (Figure S1).

In the HIV-infected sample, 75 (76%, 95%CI 66–84%) of the 99 swab cultures were positive for pneumococcus. Carriage prevalence did not vary significantly by sex, age, month of sampling or anti-retroviral treatment (p>0.2 for all, Table 2). By comparison with the population-based sample, the colonization prevalence in the HIV-infected group was higher (65.8% vs. 76%, p = 0.04). After accounting for age, month of sampling, and restricting the analysis to the period when survey of both samples was concurrent, the odds of colonization were significantly higher in the HIV-infected sample (OR 2.21, 95% CI 1.35–3.61).

**Table 2:** Carriage prevalence by age, sex and antiretroviral drug use among HIV-infected children.

Variable	Sample	Carriers	Prevalence (%)	95% CI	χ^2^ p value
Sex					
Male	68	50	74	61–83	
Female	31	25	81	63–93	0.44
Age (m)					
3–23	33	22	66	48–82	
24–41	31	24	77	59–90	
42–59	35	29	83	66–93	0.29
Antiretroviral therapy					
Yes	40	31	75	62–85	
No	59	44	78	62–89	0.74

Risk factors that were positively associated with nasopharyngeal carriage of *S. pneumoniae* were a history of coryza or cough in the previous two weeks; children who reported taking antibiotics in the previous two weeks had lower odds of carriage (Table 3, Table S1). There were no significant interactions between variables in the final model. The Hosmer-Lemeshow χ^2^ test was non-significant (df 8, p = 0.95) suggesting a good fit of the model to the data. None of the risk factors were associated with carriage in the HIV-infected sample.

**Table 3:** Risk factors for prevalent nasopharyngeal carriage of *S. pneumoniae*.

Risk factor	Sample	Carriers	OR	95% CI	aOR	95% CI
Male sex	1435	934	0.94	0.81–1.10		
Cough in the last 2 weeks?	1420	1079	2.53	2.15–2.96	1.55	1.26–1.91
Coryza in the last 2 weeks?	1684	1286	3.18	2.71–3.73	2.62	2.12–3.34
Child taken antibiotics in the last 2 weeks?	111	67	0.78	0.53–1.16	0.53	0.34–0.81
Child taken Fansidar in the last 2 weeks?	30	17	0.68	0.33–1.40		
Child hospitalized in the last month?	8	5	0.87	0.21–3.63		
Child sleeps in the cooking room?	496	344	1.22	0.99–1.50		
Cooking fuel						
firewood	2761	1814	1.00			
gas	10	8	2.09	0.44–9.85		
charcoal	60	41	1.13	0.65–1.95		
paraffin	9	5	0.65	0.17–2.44		
Smoker in the house	517	368	1.36	1.10–1.67		
Household member hospitalized in last month	18	12	1.04	0.39–2.78		
Study time	(per month)		1.00	0.99–1.01	0.98	0.96–0.99
No. of other children (aged 0–2 y) in the house	(per child)		0.82	0.71–0.95		
No. of other children (aged 3–4 y) in the house	(per child)		1.11	0.97–1.28		
No. of other children (aged5–9 y) in the house	(per child)		1.05	0.97–1.14		
No. of other children (aged 10–14 y) in the house	(per child)		1.01	0.95–1.07		
No. of children (aged ≤5 y) sharing a bed with child	(per child)		0.95	0.84–1.07		

OR Odds Ratio; aOR adjusted Odds Ratio. Variables included in the final model but not displayed here are age (6 monthly strata), month of sampling and fieldworker taking the sample (n = 9). The full analysis is displayed in Table S2. The final model fit was tested using Hosmer-Lemeshow χ^2^ in 10 covariate strata (p = 0.95).

Although the prevalence of carriage varied from month to month we did not find any evidence of a trend in prevalence over time in univariate analyses (χ^2^ test for trend by month p = 0.71). However, in the multivariable analysis of carriage, after adjusting for potential confounders, we observed a 2% decline in carriage per month of observation (Table 3).

Among the population-based sample, we isolated a total of 53 different serotypes from all the swab cultures. The carriage prevalence of individual serotypes ranged from 10% to 0.03% (Table 4, Table S2). Among HIV-infected children, we isolated a total of 23 different serotypes and carriage prevalence of any one serotype ranged from 14% to 1%. The distribution of serotypes in the population-based sample and the HIV-infected sample did not differ (χ^2^ (28) = 33.4, p = 0.22). The ten most common serotypes in the population-based sample comprised 67.5% of all isolates; in the HIV-infected sample they comprised 79% (p = 0.044) suggesting that common serotypes dominate more effectively in HIV-infected children.

**Table 4:** Serotype carriage prevalence among population-based and HIV-infected samples.

	Population-based sample		HIV-infected sample	
Serotype	N	Prevalence (%)	N	Prevalence (%)
19F	283	10.0	14	14
6A	237	8.3	13	13
6B	184	6.5	5	5
23F	117	4.1	9	9
11A	90	3.2	1	1
14	85	3.0	1	1
35B	84	3.0	2	2
23B	70	2.5	1	1
10A	56	2.0	0	0
15B	54	1.9	3	3
19A	53	1.9	1	1
9V	51	1.8	3	3
13	49	1.7	3	3
15A	49	1.7	2	2
15C	43	1.5	2	2
34	38	1.3	0	0
3	34	1.2	4	4
16F	34	1.2	0	0
18C	29	1.0	0	0
19B	25	0.9	2	2
7C	23	0.8	1	1
20	21	0.7	3	3
23A	19	0.7	1	1
21	18	0.6	0	0
35A	15	0.5	0	0
1	13	0.5	0	0
33B	13	0.5	0	0
4	12	0.4	0	0
Other types	69	2.4	4	4
All types	1,868	65.8	75	76

There is no evidence of a difference between the HIV-infected and population-based samples in the distribution of serotypes (χ^2^ (28) 33.4, p = 0.223).

**Figure 2:**
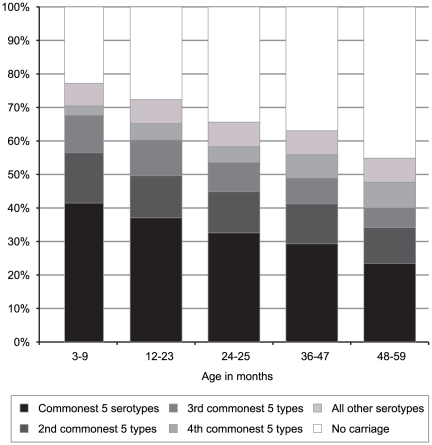
Prevalence of carriage of common and less common pneumococcal serotypes by age.

Variation in serotype prevalence by age is difficult to interpret because of the large number of strata, in this case 53 serotypes. Confining the analysis to the 20 most frequently observed serotypes, and collapsing all other types to ‘other’, an association with age in years was observed (χ^2^ (80) = 109, p = 0.017). We investigated how changes in serotype distributions with age are related to the overall prevalence of a serotype. We categorized the most highly prevalent 20 serotypes into 4 strata of 5 serotypes based on their prevalence ranking in the whole population-based survey; all remaining serotypes were categorized as ‘other’. The distribution of the prevalence of these strata against age in years is highly significant (χ^2^ (20) 106, p<0.0005, Figure 2). It illustrates a fall in the prevalence of common serotypes against age that is not matched by the mixture of less common serotypes.

The original formulation of PCV contained antigens for seven serotypes, 4, 6B, 9V, 14, 18C, 19F and 23F (PCV7). The present vaccines have extended this formulation to include serotypes 1, 5 and 7F (PCV10) and then, in addition, 3, 6A and 19A (PCV13). In the population-based sample, the proportions of all colonizing isolates that were of serotypes represented in PCV7, PCV10 and PCV13 were 41% (95% CI 38–43%), 42% (39–44%) and 59% (57–61%), respectively. In the HIV-infected sample they were 43% (31–55%), 43% (31–55%) and 67% (55–77%), respectively.

## Discussion

The prevalence of pneumococcal carriage in children in Kilifi is high – despite the insensitivity of sampling and laboratory detection methods [20], two-thirds of all children aged 3–59 months were found to be carriers. Carriage prevalence decreased steadily with age and varied significantly by season and HIV-infection. Carriage prevalence also varied by residential location though this was inherently linked to season by the rolling cross-sectional design of the study. We observed 53 different serotypes, in a well-defined human population of 250 000, maintained at widely different frequencies - a degree of diversity also observed in a similar study in The Gambia [21]. Forty-three of these serotypes, accounting for 58% of all the isolates carried, are not included in the 10-valent PCV which was introduced into the Kenyan immunization schedule in 2011.

Our estimate of pneumococcal carriage prevalence (66%) among children aged 3–59 months is similar to prevalence estimates among children aged <5 years in Pakistan (62%) [22] and Uganda (62%) [23] but lower than the prevalence in Mozambique (87%) [5], The Gambia (>90%) [24] and Malawi (84%) [25]. Each of these studies sampled sick children presenting to hospital. Estimates of carriage prevalence among healthy children were much lower, at 22% in Kenya, [26] and 48% in Malawi, [8] but even in these studies the children were selected because they attended a health facility. More recently, in a study of villages in The Gambia the prevalence was estimated at over 90% [27]. The higher prevalence of carriage among healthy children in The Gambia may be a function of the different climates, sampling in different seasons, or of differing social organization; life in The Gambia is based around villages while, in Kilifi, the population live in more widely dispersed patterns.

A key determinant of the prevalence of pneumococcal carriage in children is age. In our study carriage prevalence reached a peak of 79% in the children aged 6–11 months and then declined steadily to 51% among those aged 54–59 months. The rise between 3–5 and 6–11 months is probably attributable to cumulative exposure since birth. The subsequent decline in prevalence is attributable to an age-related increase in clearance rates. In the accompanying study [17] we observed a constant rate of acquisition from 3–59 months of age but a progressive increase in clearance rates, reducing the mean duration of a colonization episode from 45 days to 21 days. We have also shown here that the prevalence of more commonly encountered serotypes declined markedly with age but the prevalence of less common serotypes did not. This observation is compatible with acquired serotype-specific immunity, developed on the basis of prior exposure [28], [29], although to date the impact of serotype-specific immunity has been documented only for a few serotypes, and only in reducing acquisition, rather than shortening duration [28], [29], [30]. As the most common serotypes are also those with the longest durations, the same epidemiological pattern would also be predicted by species-specific immunity, such as that mediated by CD4+ Th17 cells, where the action of immune clearance occurs after a lag interval [31].

A history of upper respiratory tract infection (URTI) symptoms, cough or coryza, during the two weeks prior to nasopharyngeal sampling was strongly associated with carriage in this study as in several previous studies [6], [32], [33]. Unfortunately the cross-sectional study design does not allow us to differentiate between several explanations, which include: (1) that coryza physically enhances the trapping, adherence and growth of pneumococci in the nasopharynx; (2) that coryza may augment the sensitivity of swab sampling of the nasopharynx; (3) that pneumococcal colonization leads directly to the symptoms of an URTI; or (4) that viral URTIs and pneumococcal carriage are both independently associated with seasonal factors, such as cold weather and crowding, leading to a false association.

In Kenya, carriage prevalence has been observed to be higher among HIV-infected than among HIV-uninfected adults [34]; elsewhere pediatric serotypes (e.g. 6B, 14) have been observed more frequently in HIV-infected adults than HIV-uninfected adults [35]. Our ability to investigate these associations in children was constrained by the low prevalence of HIV-infection in Kilifi. HIV-infection is identified in 4–5% of antenatal mothers in Kilifi [36] but there is an effective program of prevention of mother-to-child transmission. Therefore, we recruited a separate sample of children, selected on the basis of known HIV-infection, and did not test our population-based sample for HIV. Although the samples were derived from different sources, and there is a small degree of misclassification in one, the study should be sensitive to large variations in epidemiology. We found HIV-infected children had a two-fold higher odds of carriage than the unselected population but we did not observe any difference in the serotype distribution of colonization with HIV prevalence.

Because of the inherent difficulties and cost of detecting multiple serotype carriage in the laboratory and the large scale of this field study, we limited our investigation to studying a single serotype per swab. This decision has a consequence in estimating the prevalence. Assuming that, in multiply colonized individuals, the probability of sampling a strain (by physical swabbing and by selection of a colony from a culture plate) is proportional to the frequency of that serotype type in that nasopharynx then the study is effectively a random sample of strains from a random sample of human hosts. This will provide an accurate estimate of the relative prevalence of different serotypes, at an ecological level, but will underestimate the absolute prevalence of colonization by each serotype in the population.

A current question of public health interest is whether the benefits of introducing PCV7 into routine childhood immunization programs in developing countries will be undermined by an increase in the incidence of non-vaccine serotype disease. The potential for ‘serotype replacement disease’ is determined by the circulation of non-vaccine serotypes and the propensity of those serotypes to cause disease once they have colonized the nasopharynx [37]. In Kilifi, 59% of carriers harbor serotypes that are not included in the 7-valent vaccine and this appears to be consistent over time in this area [6] and across the continent. Similar figures for The Gambia, Mozambique, Malawi and South Africa are in the range 51–64% [5], [7], [25], [38]. The invasive potential of these serotypes has not been evaluated in a developing country but serotypes that have been found to increase invasive disease in developed countries, e.g. 3, 7F, 15B/C, 19A, 22F, 33F and 38 [39], [40], [41], [42], [43], [44] are currently carried by 7% of all children in Kilifi.

Kenya introduced PCV10 into the routine childhood immunization program in 2011. The three additional serotypes in this formulation, 1, 5 and 7F, are important causes of invasive pneumococcal disease in Africa [4], [12] but they are carried by less than 1% of children in this study. Beyond the PCV10 serotypes we detected pneumococcal strains from 43 different serotypes accounting for 58% of all prevalent carriage episodes. Operational use of conjugate pneumococcal vaccines in Kenya, justified by strong evidence of efficacy against invasive pneumococcal disease [12], [13], will provide an interesting natural test of the invasive potential of the many non-vaccine serotypes circulating in this population.

## Supporting Information

Figure S1 **Correlation of monthly pneumococcal carriage prevalence with monthly estimates of meteorological variables.**
(PDF)

Table S1 **Prevalence of carriage by risk factor, with univariate odds ratios (and 95% CIs) and adjusted odds ratios (and 95% CIs) in a multivariable logistic regression model.**
(PDF)

Table S2 **Serotype carriage prevalence in population-based and HIV-infected samples.**
(PDF)
