# Supplementary material for: The Prevalence and Risk Factors for Pneumococcal Colonization of the Nasopharynx among Children in Kilifi District, Kenya
Source: PLoS One. 2012 Feb 20;7(2):e30787. doi: 10.1371/journal.pone.0030787 (PMC3282706; doi:10.1371/journal.pone.0030787)
Supplement: Figure S1 — Correlation of monthly pneumococcal carriage prevalence with monthly estimates of meteorological variables. (PDF) [file pone.0030787.s001.pdf]

**Figure S1. Correlation of monthly pneumococcal carriage prevalence with monthly estimates of meteorological variables.**

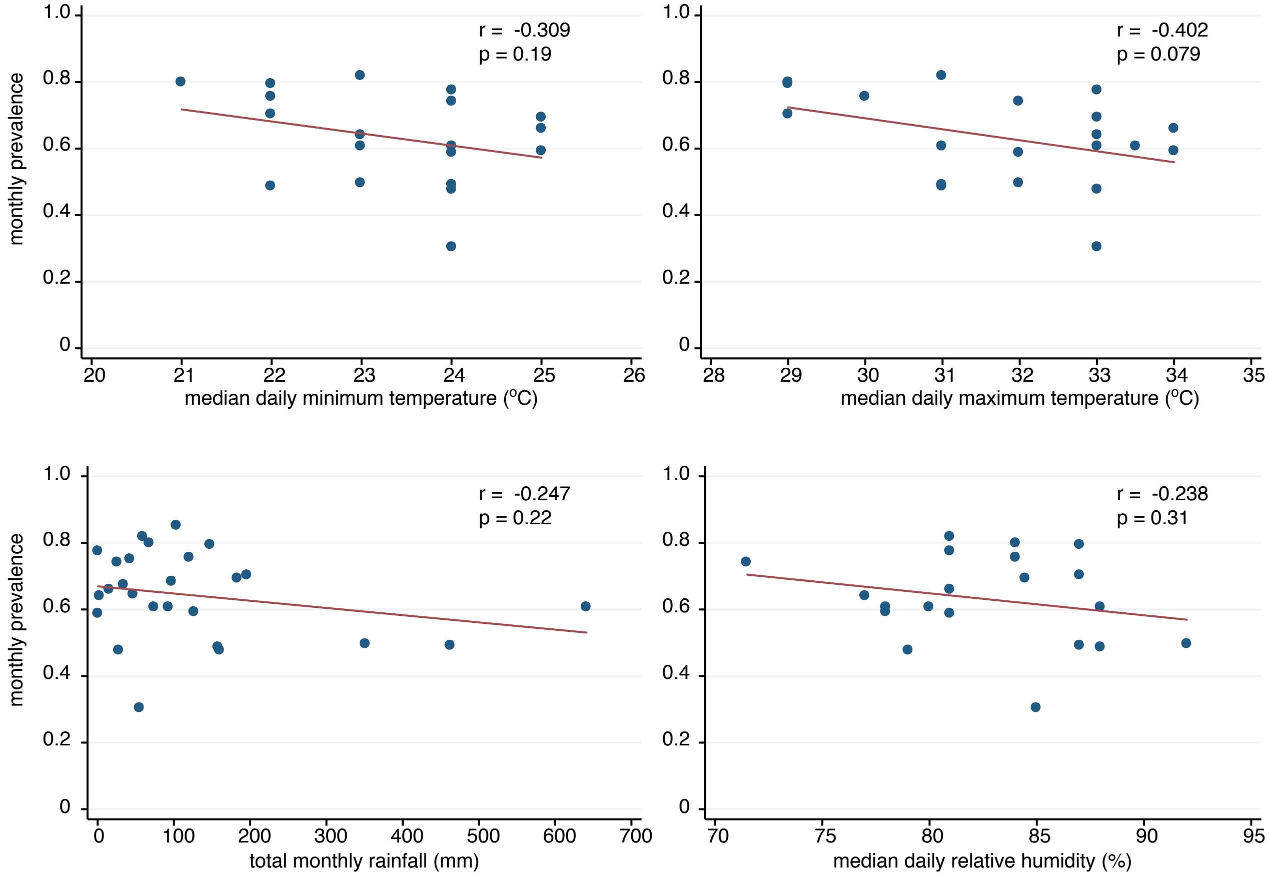


The figures show the scatter of monthly prevalence of carriage of *S. pneumoniae* in the population based sample of children aged 3-59 months against the monthly summaries of weather variables measured on a daily basis. Data on weather were available for the 26 study months (daily rainfall) or for 20 months (daily minimum/maximum temperature and daily relative humidity.)
