# Supplementary material for: The Prevalence and Risk Factors for Pneumococcal Colonization of the Nasopharynx among Children in Kilifi District, Kenya
Source: PLoS One. 2012 Feb 20;7(2):e30787. doi: 10.1371/journal.pone.0030787 (PMC3282706; doi:10.1371/journal.pone.0030787)
Supplement: Table S1 — Prevalence of carriage by risk factor, with univariate odds ratios (and 95% CIs) and adjusted odds ratios (and 95% CIs) in a multivariable logistic regression model. (PDF) [file pone.0030787.s002.pdf]

Table S1. Prevalence of carriage by risk factor, with univariate odds ratios (and 95% CIs) and adjusted odds ratios (and 95% CIs) in a multivariable logistic regression model

Exposure variable	Value	Swabs	Cultures	Prevalence	OR	95% CI	aOR	95% CI
Sex								
	female	1405	934	0.67	1.00			
	male	1435	934	0.65	0.94	0.81-1.10		
Age								
	3-5m	108	78	0.72	1.00		1.00	
	6-11m	278	220	0.79	1.46	0.88-2.43	1.33	0.77-2.28
	12-17m	304	225	0.74	1.10	0.67-1.79	0.86	0.51-1.45
	18-23m	325	230	0.71	0.93	0.57-1.51	0.86	0.51-1.43
	24-29m	300	202	0.67	0.79	0.49-1.29	0.63	0.37-1.05
	30-35m	311	199	0.64	0.68	0.42-1.10	0.60	0.36-1.00
	36-41m	277	180	0.65	0.71	0.44-1.16	0.60	0.36-1.02
	42-47m	310	190	0.61	0.61	0.38-0.98	0.54	0.33-0.9
	48-53m	319	186	0.58	0.54	0.33-0.87	0.49	0.29-0.81
	54-59m	308	158	0.51	0.41	0.25-0.65	0.37	0.22-0.62
Month of the year when sampled								
	Jan	216	110	0.51	1.00		1.00	
	Feb	278	188	0.68	2.01	1.40-2.90	1.68	1.14-2.48
	Mar	225	139	0.62	1.56	1.07-2.27	1.48	0.99-2.21
	Apr	220	140	0.64	1.69	1.15-2.47	1.85	1.23-2.80
	May	278	148	0.53	1.10	0.77-1.57	1.11	0.76-1.64
	Jun	280	193	0.69	2.14	1.48-3.09	1.86	1.25-2.78
	Jul	271	225	0.83	4.71	3.11-7.13	4.05	2.60-6.30
	Aug	118	89	0.75	2.96	1.80-4.86	2.52	1.49-4.27
	Sep	178	134	0.75	2.93	1.90-4.52	2.58	1.61-4.15
	Oct	326	239	0.73	2.65	1.84-3.80	2.25	1.52-3.34
	Nov	337	204	0.61	1.48	1.05-2.09	1.30	0.89-1.88
	Dec	113	59	0.52	1.05	0.67-1.66	0.87	0.53-1.42

Exposure variable	Value	Swabs	Cultures	Prevalence	OR	95% CI	aOR	95% CI
Has the child had cough in the last 2 weeks?								
	no	1420	789	0.56	1.00		1.00	
	yes	1420	1079	0.76	2.53	2.15-2.96	1.55	1.26-1.91
Has the child had a runny nose in the last 2 weeks?								
	no	1156	582	0.50	1.00		1.00	
	yes	1684	1286	0.76	3.18	2.71-3.73	2.62	2.12-3.34
Has the child taken antibiotics in the last 2 weeks?								
	no	2729	1801	0.66	1.00		1.00	
	yes	111	67	0.60	0.78	0.53-1.16	0.53	0.34-0.81
Has the child taken fansidar in the last 2 weeks?								
	no	2810	1851	0.66	1.00			
	yes	30	17	0.57	0.68	0.33-1.40		
Has the child been hospitalised in the last month?								
	no	2832	1863	0.66	1.00			
	yes	8	5	0.63	0.87	0.21-3.63		
Does the child sleep in the room used for cooking?								
	no	2344	1524	0.65	1.00			
	yes	496	344	0.69	1.22	0.99-1.50		
What type of cooking fuel is used?								
	firewood	2761	1814	0.66	1.00			
	gas	10	8	0.80	2.09	0.44-9.85		
	charcoal	60	41	0.68	1.13	0.65-1.95		
	paraffin	9	5	0.56	0.65	0.17-2.44		

Exposure variable	Value	Swabs	Cultures	Prevalence	OR	95% CI	aOR	95% CI
Is there a cigarette smoker in the home?								
	no	2323	1500	0.65	1.00			
	yes	517	368	0.71	1.36	1.10-1.67		
Month of study								
					1.00		1.00	
	per month				1.00	0.99-1.01	0.98	0.96-0.99
Has any co-resident been hospitalised in the last month								
	no	2822	1856		1.00			
	yes	18	12		1.04	0.39-2.78		
No. of other children (0-2y) in the household								
					1.00			
	per child				0.82	0.71-0.95		
No. of other children (3-4y) in the household								
					1.00			
	per child				1.11	0.97-1.28		
No. of children (5-9y) in the household								
					1.00			
	per child				1.05	0.97-1.14		
No. of children (10-14y) in the household								
					1.00			
	per child				1.01	0.95-1.07		
No. of children (≤5y) sharing a bed								
					1.00			
	per child				0.95	0.84-1.07		

Exposure variable	Value	Swabs	Cultures	Prevalence	OR	95% CI	aOR	95% CI
Location of residence								
	Banda ra salama	94	73	0.78	1.00			
	Chasimba	207	133	0.64	0.52	0.29-0.91		
	Jaribuni	65	38	0.59	0.40	0.20-0.81		
	Junju	282	186	0.66	0.56	0.32-0.96		
	Kauma	114	97	0.85	1.64	0.81-3.33		
	Kilifi township	269	190	0.71	0.69	0.40-1.20		
	Malindi	129	68	0.53	0.32	0.18-0.58		
	Matsangoni	239	117	0.49	0.28	0.16-0.48		
	Mtwapa	123	80	0.65	0.54	0.29-0.99		
	Ngerenya	253	168	0.66	0.57	0.33-0.99		
	Roka	251	167	0.67	0.57	0.33-0.99		
	Sokoke	137	95	0.69	0.65	0.35-1.19		
	Takaungu Mavueni	245	168	0.69	0.63	0.36-1.09		
	Tezo	256	157	0.61	0.46	0.26-0.79		
	Ziani	176	131	0.74	0.84	0.46-1.51		
Fieldworker taking the swab								
	A	408	266	0.65	1.00		1.00	
	B	11	7	0.64	0.93	0.27-3.25	0.76	0.19-3.03
	C	288	197	0.68	1.16	0.84-1.59	1.34	0.94-1.90
	D	101	65	0.64	0.96	0.61-1.52	0.89	0.53-1.52
	E	708	473	0.67	1.07	0.83-1.39	0.61	0.45-0.82
	F	109	61	0.56	0.68	0.44-1.04	0.62	0.37-1.03
	G	192	121	0.63	0.91	0.64-1.30	1.16	0.78-1.71
	H	506	332	0.66	1.02	0.77-1.34	0.84	0.62-1.13
	I	517	346	0.67	1.08	0.82-1.42	0.94	0.70-1.28

OR Odds Ratio; aOR adjusted Odds Ratio. The prevalence of carriage varied significantly by administrative location (χ^2 (14) 79, $p<0.0005$) therefore this was not included in the final model as several of the proximal causes (e.g. use of antibiotics, cigarette smoke exposure) varied markedly by location. The final model fit was tested using Hosmer-Lemeshow test in 10 covariate strata ($p=0.97$).
