# Supplementary material for: The Prevalence and Risk Factors for Pneumococcal Colonization of the Nasopharynx among Children in Kilifi District, Kenya
Source: PLoS One. 2012 Feb 20;7(2):e30787. doi: 10.1371/journal.pone.0030787 (PMC3282706; doi:10.1371/journal.pone.0030787)
Supplement: Table S2 — Serotype carriage prevalence in population-based and HIV-infected samples. (PDF) [file pone.0030787.s003.pdf]

Table S2. Serotype carriage prevalence in population-based and HIV-infected samples

	Population-based sample		HIV-infected sample	
**Serotype**	**N**	**Prevalence (%)**	**N**	**Prevalence (%)**
19F	283	9.96	14	14
6A	237	8.35	13	13
6B	184	6.48	5	5
23F	117	4.12	9	9
11A	90	3.17	1	1
14	85	2.99	1	1
35B	84	2.96	2	2
23B	70	2.46	1	1
10A	56	1.97	0	0
15B	54	1.90	3	3
19A	53	1.87	1	1
9V	51	1.80	3	3
13	49	1.73	3	3
15A	49	1.73	2	2
15C	43	1.51	2	2
34	38	1.34	0	0
3	34	1.20	4	4
16F	34	1.20	0	0
18C	29	1.02	0	0
19B	25	0.88	2	2
7C	23	0.81	1	1
20	21	0.74	3	3
23A	19	0.67	1	1
21	18	0.63	0	0
35A	15	0.53	0	0
1	13	0.46	0	0
33B	13	0.46	0	0
4	12	0.42	0	0
38	8	0.28	0	0
35F	7	0.25	1	1
10F	6	0.21	0	0
12F	4	0.14	0	0
24F	4	0.14	0	0
33D	4	0.14	1	1
10B	3	0.11	0	0
17F	3	0.11	0	0
18F	3	0.11	0	0
22A	3	0.11	0	0
22F	3	0.11	0	0
28F	3	0.11	0	0
29	3	0.11	1	1
8	2	0.07	0	0
9L	2	0.07	0	0
12B	2	0.07	0	0
5	1	0.04	0	0
7F	1	0.04	0	0
11D	1	0.04	0	0
15F	1	0.04	0	0
19C	1	0.04	0	0
28A	1	0.04	0	0
31	1	0.04	0	0
33C	1	0.04	0	0
40	1	0.04	0	0
9N	0	0	1	1
All types	1868	65.8	99	76
